# Injectable multifunctional hydrogel containing Sphingosine 1-phosphate and human acellular amniotic membrane for skin wound healing

**DOI:** 10.22038/IJBMS.2024.76681.16607

**Published:** 2024

**Authors:** Shaghayegh Doudi, Mohammad Kamalabadi-Farahani, Amir Atashi, Jafar Ai, Danial Cheraghali, Sepehr Zamani, Majid Salehi

**Affiliations:** 1 School of Medicine, Shahroud University of Medical Sciences, Shahroud, Iran; 2 Tissue Engineering and Stem Cells Research Center, Shahroud University of Medical Sciences, Shahroud, Iran; 3 Department of Tissue Engineering, School of Medicine, Shahroud University of Medical Sciences, Shahroud, Iran; 4 Department of Hematology, School of Allied Medical Sciences, Shahroud University of Medical Sciences, Shahroud, Iran; 5 Department of Tissue Engineering and Applied Cell Sciences, School of Advanced Technologies in Medicine, Tehran University of Medical; 6 Sciences, Tehran, Iran; 7 Department of Mechanical Engineering, New Jersey Institute of Technology, New Jersey, United States of America (USA); 8 Health Technology Incubator Center, Shahroud University of Medical Sciences, Shahroud, Iran; 9 Sexual Health and Fertility Research Center, Shahroud University of Medical Sciences, Shahroud, Iran

**Keywords:** Amnion, Gelatin, Hydrogels, Sodium carboxymethylcellulose, Sphingosine 1-phosphate, Wound healing

## Abstract

**Objective(s):**

The skin serves as the main defense barrier, protecting against injuries, and preventing infection and water loss. Consequently, wound healing and skin regeneration are crucial aspects of wound management. A novel hydrogel scaffold was developed by incorporating carboxymethyl cellulose (CMC) and gelatin (Gel) hydrogels cross-linked with 1-(3-dimethylaminopropyl)-3-ethylcarbodiimide hydrochloride (EDC) containing Sphingosine 1-phosphate (S1P). This hydrogel is applied topically to treat acute wounds and is covered with a human acellular amniotic membrane (hAAM) as a secondary dressing.

**Materials and Methods:**

The scaffold was subjected to *in vitro* cell viability, red blood cell hemolysis, blood clotting index, and *in vivo* assays. Real-time PCR was implemented to verify the expression of genes involved in skin wounds. The physical and chemical properties of the scaffolds were also tested using weight loss, swelling ratio, scanning electron microscopy (SEM), Fourier transform infrared (FTIR), and mechanical tensile analysis.

**Results:**

The synthetic scaffold is biocompatible as evidenced by the high percentage of 3T3 cell viability (127%) after 72 hr. Additionally, excellent hemocompatibility with a low hemolytic effect (2.26%) was observed. Our* in vivo *wound healing assay demonstrated that CMC/Gel/S1P/hAAM wound dressing led to faster wound healing in treated rats compared to the control group over 14.

Also, the mechanical tests showed that the amniotic membrane and the hAAM had very different Young’s modulus and elongation at break values.

**Conclusion:**

This study demonstrates the effectiveness of the CMC/Gel/EDC hydrogel with S1P as a wound dressing. Additionally, hAAM exhibits excellent characteristics as a protective layer for the treatment of acute wounds.

## Introduction

The skin is the largest peripheral organ in our body, which covers 8% of the whole body mass and is the obstruction between the inner and outside environment of the body (1). It is important for protecting the body from germs, sensing touch, and regulating temperature. However, because the skin is sensitive to injury, it needs proper care to heal wounds (2). The healing process involves different stages, including hemostasis, inflammation, granulation, and remodeling, and it is important to take care of wounds to prevent infection, speed up healing, reduce pain, and avoid scarring (3). Although autograft and allograft treatment strategies are usually effective in most cases, the lack of donor resources and re-surgery, and how to solve the problem of covering large wound areas and reducing the scarring effect in patients with huge wounds or those facing cosmetic demands are still a clinical challenge (4). Recently, the development of tissue engineering technology, which includes three main elements; cells, growth factors, and scaffolds; has made the treatment of many different diseases easier (5).

Wound dressings have become important in the medical field and can be used to deliver drugs and other substances to wounds. They can be used for targeted delivery of drugs, antibiotics, nanoparticles, growth factors, and regulatory peptides (3). There are different types of wound dressings, including gauze, foam, film, hydrocolloid, and hydrogels. Hydrogels are commonly used because they can retain moisture and mimic natural tissue. They can also promote cell growth and offer a cooling effect (6). One of their key features is that they can absorb liquid without losing their shape. The amount of liquid they can absorb can be adjusted by changing their concentration and density (7). Previous studies discuss the different choices of natural and synthetic polymers used in making hydrogels (7). Gelatin is one such natural material with various functional properties and applications (8). Gelatin is derived from collagen and has biocompatibility, biodegradability, and bioactivity. It can be made into different forms for different purposes. Due to its cell adhesion domains, gelatin is commonly used in tissue engineering and wound dressing applications (9). Another advantage of gelatin is that it and the product after its degradation are both non-toxic (10). Carboxymethyl cellulose (CMC) is a natural material that is water-soluble and hydrophilic. It is commonly used in pharmaceutical and biomedical applications, particularly in wound dressings. These dressings are flexible, absorbent, and promote healing (11). CMC is non-toxic and abundant, making it a good choice for medications or implantation in the body (11). CMC-based hydrogels help wound healing because they maintain a moist environment around the wound (11). When gelatin and CMC are combined, they can form stable hydrogels (7). Using crosslinkers like 1-(3-dimethylaminopropyl)-3-ethylcarbodiimide hydrochloride (EDC) can make these hydrogels even more stable, which makes them better for tissue engineering(12). EDC also has low cytotoxicity and can crosslink hydrogels without being directly involved in the bonds (12).

Previous studies discuss the importance of bioactive molecules in wound dressings and how they can be effectively delivered to the affected area (13). In particular, Sphingosine 1-phosphate (S1P) as a potent growth factor is involved in the regulation of cell proliferation, differentiation, survival, and motility, and by increasing its intracellular content through sphingolipid metabolism and binding to its receptors, it regulates a number of physiological/pathological processes. Among them, autophagy, migration, angiogenesis, and the formation of new vessels can be mentioned (14). For these reasons, as a bioactive lysophospholipid, sphingosine 1-phosphate (S1P) is very important for wound healing because it helps cells grow, survive, and move around(15, 16). S1P contributes to early wound healing by regulating the integrity of the endothelial layer barrier, inhibiting smooth muscle cell migration (15-17), and preventing excessive skin tissue proliferation (18-21). Moreover, incorporating S1P into a hydrogel can enhance tissue regeneration and make it a promising therapeutic molecule in wound management (18).

The human acellular amniotic membrane (hAAM), a cost-effective and accessible option for secondary wound dressings (22), has been studied in reconstructive surgery and wound healing since 1910. hAAM is a thin and transparent membrane with multiple layers, including active epithelium, basement membrane, compact layer, fibroblast layer, and spongy layer. Decellularized hAM would prevent sensitization and immune reactions, reduce infection risk, and lower costs compared to cellularized grafts (23-27). hAAM exerts anti-infection effects by producing anti-inflammatory proteins and reducing certain pro-inflammatory cytokines (24, 28).

Although the effect of each component used in this project has been investigated separately in previous studies, the effect of a two-layer wound dressing on accelerating wound healing and skin regeneration has not been investigated. Also, the optimal amount of EDC for proper cross-linking of CMC/Gel with the lowest level of toxicity and the appropriate concentration of these materials for hydrogel synthesis was determined. In this study, gelatin and CMC with a high degree of biocompatibility and biodegradability, are crosslinked with EDC. This copolymerized hydrogel carries and releases S1P topically at the wound site. hAAM was also used as a natural secondary wound dressing to cover the injected hydrogel.

## Materials and Methods

**Materials**

Carboxymethyl cellulose (CMC), Gelatin (Gel), Sphingosine 1-phosphate (S1P), penicillin, streptomycin, methanol, and 1-(3-dimethylaminopropyl)-3-ethylcarbodiimide hydrochloride (EDC) purchased from Sigma-Aldrich (St. Louis, USA). 3-(4, 5- dimethylthiazol-2-yl)-2,5 diphenyl tetrazolium bromide (MTT), Dulbecco’s modified Eagle’s medium: nutrient mixture F-12 (DMEM/F12) and fetal bovine serum (FBS) bought from Gibco, BRL (Eggenstein, Germany).

**Human amniotic membrane retrieval and decellularization**

In accordance with the policy of Shahroud University of Medical Sciences and Bahar Hospital, amniotic membranes were isolated from the placenta of healthy mothers who gave birth by cesarean section, to prevent any possible contamination during natural delivery. The placenta was transferred to the laboratory within a sterilized ice bag containing ethylene oxide (SUPA medical co.), 0.9% normal saline 1% penicillin, 1% streptomycin, and 2.5 μg/ml amphotericin B. To remove extra tissues, blood, and contaminations, the placenta was washed with PBS and the above-mentioned antibiotics three times in an aseptic laboratory. Then amniotic membrane was separated from chorion and other components of the placenta and washed carefully with sterile PBS.

A chemical-physical approach was performed for decellularization. First, the amniotic membranes were immersed in 100 ml of 2% EDTA and incubated at 37 °C for 30 min. Then, they were immersed in 50 ml of 0.5 M NaOH, and 50 ml of 5% ammonium chloride, subsequently, while shaking for 5 min. Next, the tissue was spread on a sterile aluminum foil and drawn with a sterile gauze soaked in PBS to physically scrape the surface. After three more washes, it was stored at -20 °C for long-term use.

***hAAM characterization***

*DNA content*

To assess the cellular and DNA residual from tissue samples, DNA was extracted following Pouya Gene Azma kit. DNA absorbance was measured using a Biotek cytation 5 plate reader/image reader at 260 and 280 nm. 

*Electrophoresis*

To verify the success of the DNA extraction procedure, an electrophoresis assay was conducted. 9 μl of DNA extracted from decellularized amniotic membranes loaded into the wells of 1% agarose gel and 1 ml of green viewer DNA. The gel was run for 20 min and then analyzed using a UVitec machine.

*H* *&* *E staining*

Tissues are fixed in 10% (w/v) formalin and embedded in paraffin wax. Sections were then cut at 5-micron thicknesses using a microtome and stained with hematoxylin and eosin to visualize collagen and cell nuclei.

*DAPI Staining:*

DAPI staining was performed to observe the nuclei under fluorescent microscopy (Olympus 1x71; Tokyo, Japan). For this purpose, after washing hAAM several times with PBS and fixing it on the slide, it was exposed to DAPI dye, then after 10 min, it was visualized using a fluorescence microscope with an excitation source of violet light. When DAPI binds with double-strand DNA, the largest excitation wavelength is 360 nm and the largest emission wavelength is 460 nm.

*Scanning electron microscope:*

The freeze-dried hydrogel samples and decellularized human amniotic membranes (hAAM) were cut into thin layers with a diameter of 8 mm and then sputter-coated with gold (SC7620, Quorum Technologies, England) for 300 sec. The morphology of the samples was evaluated using scanning electron microscopy (FE-SEM (MiRa3–TE scan, Czech Republic) at a 20 kV accelerating voltage. The average pore size was calculated by analyzing 10 randomly chosen points per image via Image J software (National Institute of Health, Bethesda, USA) and Origin Pro 2015 software (Origin Lab, Northampton, USA).

*Tensile strength*

The mechanical properties of decellularized amniotic membranes were evaluated using a uniaxial tensile machine (DBBP-200, Santam, Korea) at room temperature. Specimens (4 cm×1 cm pieces) were subjected to a maximum load of 5 kN and extended at a rate of 2 mm/min. After reaching the fracture point, the stress-strain curve was plotted. The gradient of the initial portion of the stress-strain graph indicates Young’s Modulus of each tissue.

**Hydrogel fabrication**

**Figure 1:**
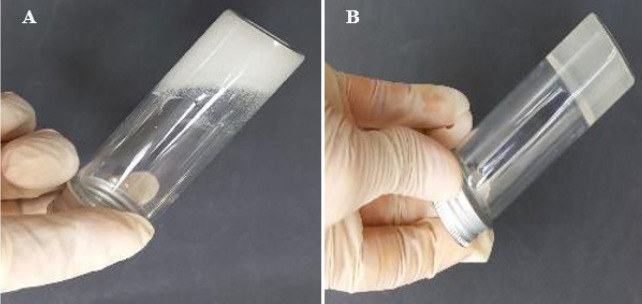
CMC/Gel hydrogel without crosslinker (A) and CMC/Gel hydrogel crosslinked with EDC (B)

The carboxymethyl cellulose and Gelatin hydrogels were produced by dissolving 3% (W/V) of each polymer in ddH_2_O for 24 hr. Then, they were mixed at the ratio of 4:1 CMC/Gel and homogenized for 24 hr. Next, different percentages (0.01, 0.05, 0.1, 0.5, and 1% W/V) of 1-(3-dimethylaminopropyl)-3-ethylcarbodiimide hydrochloride were added dropwise to the solution to achieve the appropriate mechanical strength required for the hydrogel, followed by 4 hr stirring at ambient temperature (Figure 1). Simultaneously, different doses of S1P (1, 5, 10, 20, 40, and 80 μl) were added to the prepared hydrogel with the optimum EDC crosslinker and stirred for another 24 hr at RT.

***Lyophilization***

After freezing the hydrogel at -80 °C for 24 hr, they become lyophilized at -54 °C for 48 hr in a freeze-dryer device (Telstar, Terrassa, Spain).

***Hydrogel Characterization***

*Fourier Transform Infrared (FTIR)*

We utilized FTIR Spectroscopy (Spectrum GX, PerkinElmer, USA) to identify the chemical structures within the scaffolds. The technique was applied within a range of 4000-400 cm^-1^.

*Weight loss*

The degradation rate of hydrogels was evaluated by measuring the mass loss. After weighing three replicates of each group, the tissues were placed in a falcon tube containing PBS at 37 °C for 24 and 48 hr. The samples were dried after removing from the solution. The degree of degradation was calculated using equation 1, where W_0_ represents the initial weight of lyophilized hydrogels and W_1_ is the dry weight after removal from the solution.

$\mathrm{Weight loss\%}=\frac{\left(W_{0}-W_{1}\right)}{W_{0}}\times 100$

Eq. 1

*Swelling ratio*

The capacity to uptake the liquids is essential for the proper function of wound dressings. The water absorption capability of hydrogels was determined by first weighing the dry mass and then soaking them in PBS at RT for different periods (1, 3, 6, 12, 24, and 48 hr). The percentage of water absorption capacity was quantified using equation 2, where W_d_ denotes the initial weight of lyophilized hydrogels and W_s_ is the wet weight after removal from the solution.

$\mathrm{Swelling Ratio\%}=\frac{\left(W_{s}-W_{d}\right)}{W_{d}}\times 100$

Eq. 2

*Blood compatibility*

In this experiment, 2 ml of fresh human blood containing an anticoagulant was diluted with 2.5 ml of 0.9% normal saline. One hundred microliters of each hydrogel with different concentrations of S1P were placed in the well of a 96-well plate, to which 0.2 ml of the diluted blood was added until it covered up the hydrogels. The plate was incubated at 37 °C for 60 min and then centrifuged at 1500 rpm for 10 min. The absorbance of the supernatant was measured at 545 nm using a Biotek microplate reader (USA).

Negative control of 0.2 ml blood/4 ml normal saline, and positive control of 0.2 ml blood/4 ml ddH_2_O were included in this experiment. Subsequently, the extent of hemolysis was determined using the following equation (Eq. 3):

$\mathrm{Hemolysis\%}=\frac{\left(D_{t}-D_{\mathrm{nc}}\right)}{\left(D_{\mathrm{pc}}-D_{\mathrm{nc}}\right)}\times 100$

Eq. 3

Where D_t_ represents the sample absorption, D_nc_ denotes the negative control absorption, and D_pc_ signifies the positive control absorption.

*Blood clotting index*

One milliliter of each concentration of hydrogel was added to a 25 ml beaker and incubated in a water bath at 37 °C for 1 hr. Subsequently, 100 µl of blood containing anticoagulant was added to the hydrogels, followed by adding 20 µl of 0.2 M CaCl_2_ after 5 min. After an additional 5 min, 25 ml ddH_2_O was slowly added. Then, the absorbance of 100 µl supernatant was measured at 545 nm. ddH_2_O with anticoagulated blood was included as a control. Finally, the results were calculated using the following equation (Eq. 4):

$\mathrm{Blood Clotting Index \%}=\frac{A_{\mathrm{Sample}}}{A_{\mathrm{Control}}}\times 100$

Eq. 4

The amount of sample absorption is represented by A_Sample_, whereas A_Control_ stands for the control absorption value.

*MTT test*

In this study, 3T3 fibroblast cells were cultured in DMEM with high glucose, 10% FBS, and 1% penicillin/streptomycin antibiotic. 3-(4,5-dimethylthiazol-2-yl)-2,5-diphenyl-2H-tetrazolium bromide (MTT) assay used to measure the cell viability. In this experiment, DMEM poured on the hydrogel and the extract was collected after 24 and 72 hr for further analysis. Cultured 3T3 fibroblast treated with hydrogel extract for different time points. A control group without the hydrogel was also included. After incubation time, MTT solution added to each well and the plate incubated at 37 °C for 4 hr. The resulting solution was measured using a microplate reader. This experiment was repeated three times for each sample. Equation 5 was also used to calculate the survival rate:

$\mathrm{Cell Viability \%}=\frac{{\mathrm{OD}}_{\mathrm{Treatment}}}{{\mathrm{OD}}_{\mathrm{Control}}}\times 100$

Eq. 5

Where OD_Treatment_ and OD_Control_ denote the average value of the measured optical density of samples with and without hydrogels, respectively.

***In vivo*** **study**

In this study, a full-thickness excisional wound model was conducted in accordance with the arrival guidelines and was approved by the ethics committee of Shahroud University of Medical Sciences (IR.SHMU.REC.1401.013) to assess the wound healing efficacy of a CMC/Gel hydrogel+S1P covered with hAAM. Thirty adult male Wistar rats (2 months old, weighing 200–250 g) were used in the experiment. Briefly, a full-thickness excisional wound (1.50×1.50 cm^2^) was created on the back skin of rats under general anesthesia with ketamine (100 mg/kg body weight) and xylazine (10 mg/kg body weight). Five experimental groups were established: positive control, negative control (treated with sterile gauze), CMC/Gel/hAAM, CMC/Gel/S1P/hAAM, and hAAM groups. The hAAM covering the wounds was sutured diagonally at two opposite corners and secured with adhesive bandages for further protection. The percentage of wound closure was assessed using equation 6, where stands for the wound area on the surgery day and denotes the area of the wound on the desired day.

$\mathrm{Wound Closure\%}=\frac{A_{\mathrm{Surgery}}-A_{\mathrm{Wound}}}{A_{\mathrm{Surgery}}}\times 100$

Eq. 6

***RNA extraction***

RNA was extracted from skin tissue according to an RNA extraction kit (Parstous Company, Iran). Briefly, skin tissues were crushed and immersed in Trizol, then mixed with chloroform and centrifuged. After several additional steps, the concentration of extracted RNA was measured by plate reader/image device reader Biotek model cytation 5 at 260 and 280 nm. Extracted RNA was stored at -80 °C.

*cDNA synthesis*

cDNA was made from extracted RNAs (5 ng) following the protocol provided in the Parstous kit. Briefly, RNA was combined with 10 µl Buffer-Mix, 2 µl Enzyme-mix, and up to 20 µl DEPC-treated water. Then, it was incubated at specific temperatures and the reaction was stopped by heating. The final product was cooled on ice at 4 °C.

*Real-time PCR*

Real-Time PCR technique was used to evaluate the expression levels of transforming growth factor-beta 1 (TGF-β1), Insulin-like growth factor (IGF-1), VERSICAN (VCAN), COLLAGEN-1, and vascular endothelial growth factor (VEGF-α) genes in relation to the housekeeping GAPDH gene. Primer sequences are provided in Table 1. Roche Light-Cycle 96 and SYBR Green were used as fluorescent substances, and a standard diagram was made to check the efficiency of the reaction) Table 1).

***Histopathological examination***

7- and 14-days post-treatment, the animals were euthanized with ketamine (200 mg/kg body weight) and xylazine (20 mg/kg body weight), and skin tissues were harvested and fixed in formalin (10%, pH:7.26) After 48 hr, tissues were sectioned into 5 μm slices and stained with H&E and Verhoeff-Van Gieson (VVG) staining. An independent reviewer evaluated the epithelialization, angiogenesis, fibroplasia, and granulation tissue formation among the different groups using light microscopy (Olympus BX51; Olympus, Tokyo, Japan).

*Histomorphometric analysis*

On day 14, the degree of re-epithelialization was assessed semi-quantitatively using a 5-point scale (0=no re-epithelialization, 1=25%, 2=50%, 3=75%, and 4=100%), and the results were double checked by an independent observer in a blinded manner.

***Statistical analysis***

Statistical analysis was conducted using GraphPad Prism 9.4 software (Minitab Inc., State College, USA) with a two-way analysis of variance. The data was collected and repeated three times, with the mean±standard deviation (SD) reported. Statistical significance was determined at *P*<0.05.

## Results

***Characterization of decellularized hAM***

**Figure 2:**
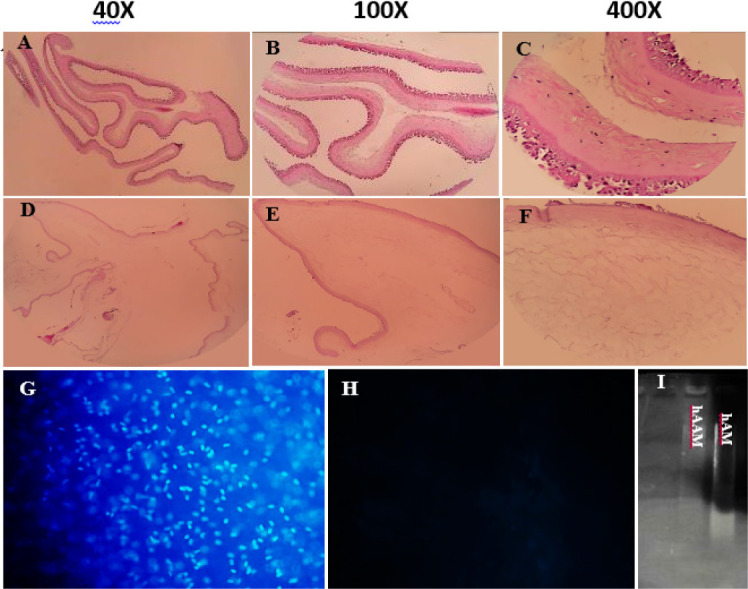
H&E staining of human amniotic membrane (A, B, and C), and decellularized human amniotic membrane (D, E, and F), DAPI staining decellularized human amniotic membrane (G), human amniotic membrane (H), and Electrophoresis (I)

To investigate the decellularized hAM, we performed DNA content analysis, gel electrophoresis, H&E, and DAPI staining (Figure 2). The absence of residual nuclei in H&E (A-F) and DAPI (G, H) staining was demonstrated by a lack of positive staining after the decellularization process. This finding was further confirmed by DNA quantification which revealed a significant decrease in the amount of residual DNA in hAAM (<50 ng/mg of dry tissue) compared to non-treated hAM and hAAM (1620 and 3.15 ng/mg respectively, *P*<0.05). Gel electrophoresis (I) of decellularized tissue also showed no visible residual DNA after the decellularization process (29).

*Morphological aspects and mechanical tensile*

**Figure 3:**
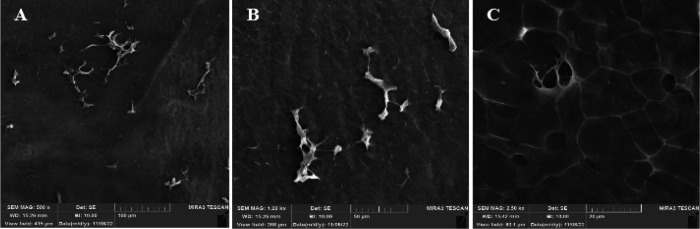
SEM of decellularized human amniotic membrane; A(X500), B(X1000), and C(X2500)

To assess the integrity of the microscopic structure of the decellularized amniotic membrane, a portion of tissue that underwent a freeze-drying procedure was examined using SEM. The results demonstrated that the tissue maintained its integrity following the decellularization procedure (Figure 3 A-C). To further determine the tensile strength of the decellularized tissue, a Universal Testing Machine was employed. Both decellularized and normal tissues were subjected to tension at a constant rate of 2 mm/min. Table 2 shows that the decellularized amniotic membrane had a much higher tensile strength than the control sample, and its structure did not change much after it was decellularized.

***Optimization of crosslinker EDC***

*Hydrogel weight loss test*

**Figure 4:**
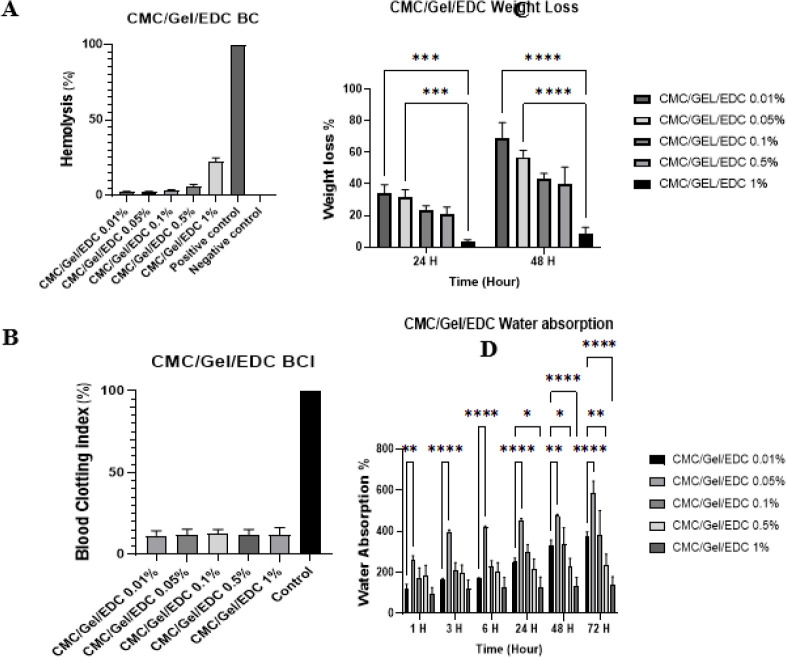
Weight loss (A), water absorption (B), blood compatibility (C), and blood clotting index (D) of hydrogel scaffolds with and without EDC crosslinker

As the degradability of hydrogel can facilitate the release of active substances in the process of wound healing, we performed a hydrogel weight loss test to investigate the biodegradability of CMC/Gel hydrogel in various concentrations (Figure 4A). First, the CMC/Gel hydrogel structure was produced by combining different concentrations of EDC to control the rate of biodegradability during wound healing. The primary and secondary weights of hydrogels were calculated (equation 1) 24 and 48 hr after EDC treatment (Figure 4A). Our data demonstrated that the degradability of hydrogel groups increased by approximately 9, 40, 43, 57, and 69 percent, respectively, after 48 hr of treatment with different concentrations (1, 0.5, 0.1, 0.05, and 0.01 (%V/V)) of EDC. These findings suggest that lower concentrations of EDC are more appropriate to achieve optimal degradability of hydrogel.

*Swelling ratio test*

It is crucial for hydrogel to cover the affected area, enhance cell growth and migration, absorb secretions, and maintain moisture. To examine the water absorption of CMC/Gel hydrogel with different concentrations of EDC, the swelling ratio was calculated by measuring the weight of each scaffold using Equation 2 (Figure 4B). The results of this experiment revealed that water absorption increased by 135%, 235%, 378%, 585%, and 371% over 72 hr in the groups with EDC concentrations of 1%, 0.5%, 0.1%, 0.05%, and 0.01%, respectively. Additionally, the swelling ratio initially experienced a slight increase upon the addition of crosslinker ranging from 0.01% to 0.05%, but subsequently declined as more crosslinker was added to the hydrogel. Since water absorption was acceptable across all concentrations, the lowest EDC concentration with minimal cytotoxicity was selected for further analysis. 

*Blood compatibility test*

The initial phase of interaction between hydrogel and red blood cells (RBCs) is considered to be a fast phenomenon, following the body’s response to inflammation. The hemolysis degree of RBCs in direct contact with hydrogel and drugs is a determinant factor of hemocompatibility. Hemolysis refers to the amount of hemoglobin released in the bloodstream as a result of damage to RBCs which has been found to have an inverse relationship with blood compatibility for different substances. Based on the hemolysis test, we found that concentrations of 1% and 0.5% were unsuitable as they caused an increased hemolysis rate of above 5%, whereas concentrations of 0.1%, 0.05%, and 0.01% were deemed more appropriate by having hemolysis rate of less than 5% (Figure 4C).

*Blood clotting index test*

Platelet aggregation and blood coagulation tests are significant components of the homeostasis process. The blood coagulation test serves as an effective method to assess the antithrombotic activity of biomaterials. A lower Blood Clotting Index (BCI) in the blood coagulation test indicates improved wound dressing structure. To further assess the anti-thrombotic function of our hydrogel, the CMC/Gel hydrogel treated with different concentrations of EDC was subjected to a BCI test. As depicted in Figure 4, all different concentrations of EDC showed significantly lower BCI rates compared to the control group, ranging from 11% to 12%. These findings suggest that these hydrogel structures are promising in the wound healing process as they do not have the potential to promote blood clot formation (Figure 4D).

*Cell viability test*

**Figure 5:**
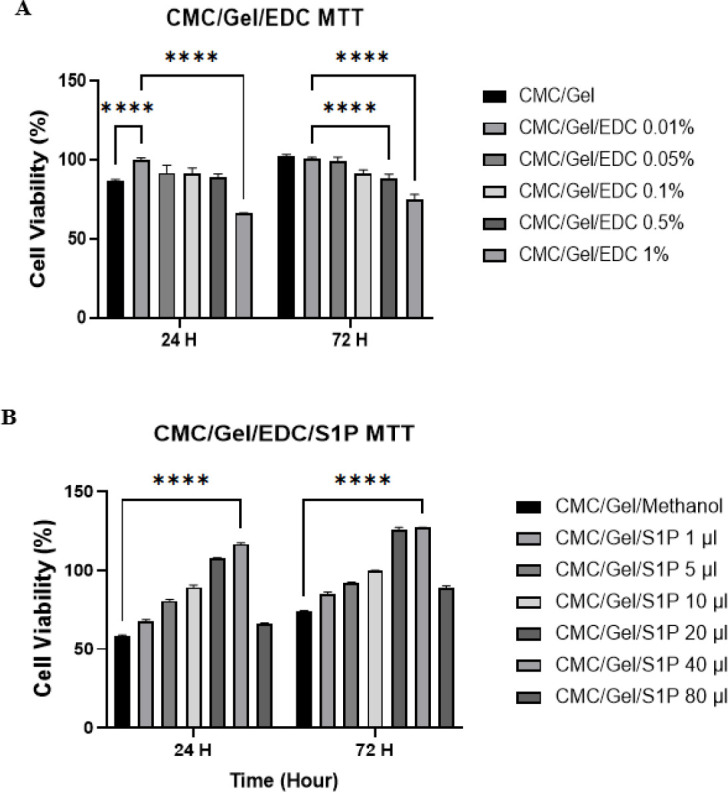
MTT assay of CMC/Gel/EDC hydrogel scaffolds containing different percentages of EDC (A) and MTT assay of CMC/Gel/EDC hydrogel scaffolds containing different percentages of S1P (B)

The appropriate concentration of EDC for cross-linking the scaffold was determined by MTT cell viability assays of 3T3 fibroblast cells treated with CMC/Gel hydrogel containing different concentrations of EDC crosslinker (1%, 0.5%, 0.1%, 0.05%, and 0.01%) for 24 and 72 hr. As shown in Figure 5, the proliferation rate of 3T3 cells on CMC/Gel hydrogel+0.01% EDC was 99.9% after 24 hr. There was also an increase in the percentage of live cells (100.98%) treated with hydrogel+0.01% EDC compared to the control group after 72 hr, while there was not a significant rise in cell survival at higher doses of 0.1%, 0.5%, and 1% of EDC (Figure 5A). Although the survival rate of cells improved after 72 hr of treatment with CMC/Gel hydrogel without a crosslinker, it is necessary to add the lowest amount of EDC to the hydrogel structure to make it more stable.

Upon conducting the aforementioned tests and verifying the most appropriate concentration of EDC (0.01%), the optimum hydrogel was formulated. This concentration not only generated improved outcomes but also enhanced the stability and minimized the cytotoxicity.

***Optimization of S1P concentration***

*Cell viability test*

To determine the optimal concentration of S1P in the scaffold structures, the MTT assay was applied. Here, 3T3 fibroblast cells were exposed to different concentrations of S1P (1, 5, 10, 20, 40 and 80 µl of stock solution (1 mg S1P/500 µl methanol) in CMC/Gel hydrogel for 24 and 72 hr. As shown in Figure 5B, cell viability increased in the presence of S1P dose-dependently (up to 40 µl) compared to CMC/Gel hydrogel without S1P, but there was a significant decrease when the concentration reached 80 µl. 

*Weight loss analysis*

**Figure 6:**
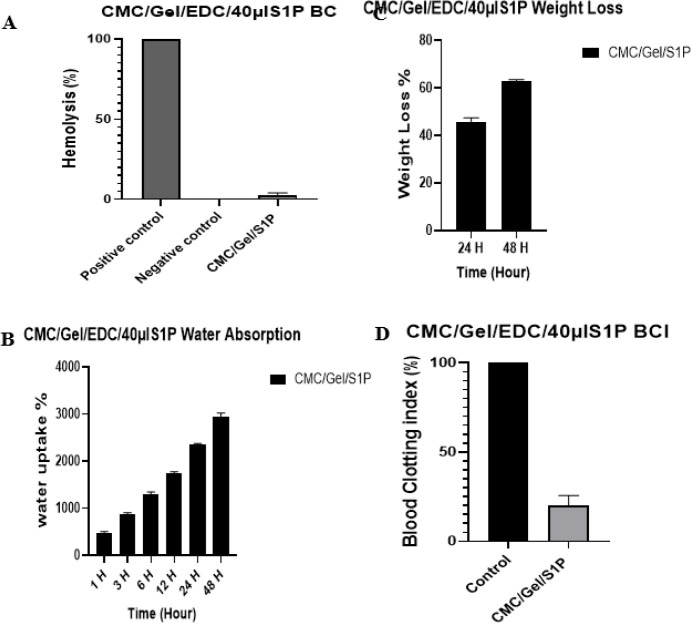
Weight loss (A), water absorption (B), blood compatibility (C), and blood clotting index (D) analysis of CMC/Gel/EDC hydrogel containing 40 µl S1P

CMC: Carboxymethyl cellulose; Gel: Gelatin; EDC: 1-(3-dimethylaminopropyl)-3-ethylcarbodiimide hydrochloride; SIP: Sphingosine 1-phosphate

After calculating the initial and secondary weights (using equation 1) of CMC/Gel scaffolds+40 µl S1P at 24 and 48 hr, respectively, we found that integration of S1P into the hydrogel increased weight loss by approximately 18% as demonstrated in Figure 6A.

*Swelling ratio analysis*

The porous structure of the material contributes to its efficacy in absorbing water. Data presented in Figure 6B indicate that CMC/Gel hydrogel containing 40 µl of S1P had fluid absorption up to thirty folds of its initial weight after 48 hr. 

*Blood compatibility (BC) and blood clotting index (BCI)*

The blood compatibility and coagulation rate of CMC/Gel hydrogel with 40 µl S1P were observed at 2.26% and 19.86%, respectively (Figures 6C and 6D).

*Morphological aspect*

**Figure 7:**
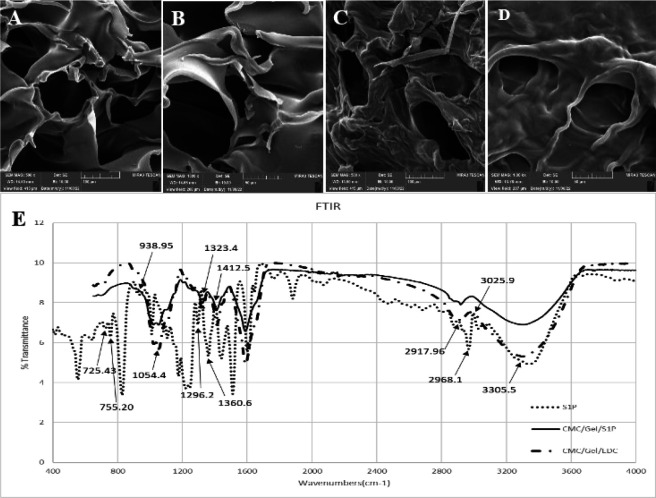
SEM of CMC/Gel/EDC hydrogel scaffolds without (A: X500, B: X1000) and with S1P (C: X500, D: X1000), and FTIR analysis of S1P powder, CMC/Gel/EDC hydrogel, and CMC/Gel/EDC hydrogel containing S1P (E)

CMC: Carboxymethyl cellulose; Gel: Gelatin; EDC: 1-(3-dimethylaminopropyl)-3-ethylcarbodiimide hydrochloride; SIP: Sphingosine 1-phosphate; FTIR: Fourier Transform Infrared

SEM imaging was used to determine the effects of S1P on the structure of CMC/Gel hydrogel. The SEM images revealed highly porous structures with interconnected pores in the inner structure of the hydrogels, which make them suitable for cell attachment and migration (Figure 7A, B, C, D). The pores were generated through phase separation of the lyophilization procedure. The size of pores in the hydrogel without S1P was in the range of 111.40 and 37.71 mm, along with S1P. These interconnected pores within the hydrogel provided a porosity of 37% and 20% for the CMC/Gel and CMC/Gel/S1P hydrogels, respectively. 

*Fourier transforms infrared spectroscopy (FTIR) analysis*

The interaction of functional groups in a CMC/Gel hydrogel with optimal EDC with and without S1P, as well as S1P powder alone, was investigated by recording the FTIR spectrum in the range of 4000 to 400 cm^-1^ (Figure 7E). FTIR analysis identified the peaks at 3305.58, 2917.96, 1412.58, 1323.46, and 1054.46 related to hydrogen bonds/hydroxyl groups, methylene/aliphatic groups, hydrocarbon groups, amide bonds, and amine groups, respectively. The presence of a hydroxyl group in the range of 1630 to 1680 is associated with the amide bond. Moreover, spectroscopic analysis revealed the presence of various peaks in the range of 3025.95 for CH groups, 2968.14 for methyl groups, 1360.67 for amine groups, 1296.24 for organic phosphate and hydroxyl groups, 938.95 for phosphate, 755.20 for carbon bonds, and 725.43 for the methylene group of sphingosine phosphate. The presence of these peaks suggests the potential involvement of CMC, GEL, and EDC in making connections with S1P. Additionally, we observed a coincidence of the peaks between S1P and synthesized hydrogel. This indicates that our prepared product contains S1P.

***hAAM implantation improved wound healing in in vivo model***

**Figure 8:**
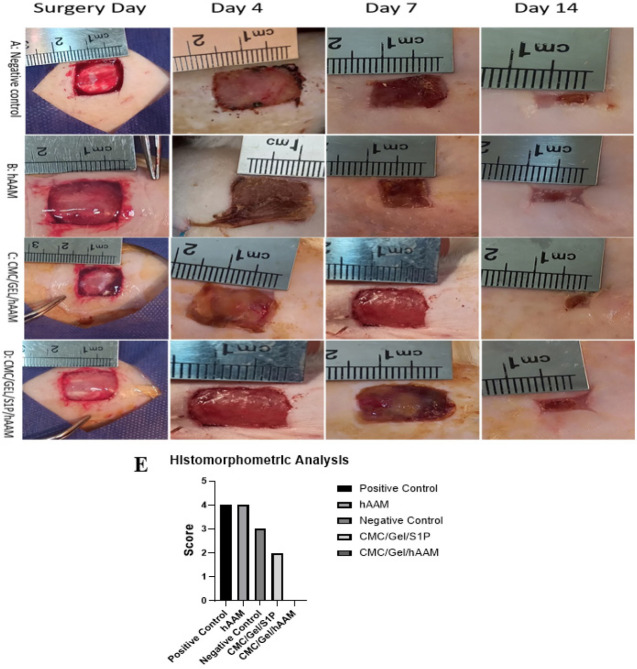
*In vivo *studies of Negative control (A), hAAM (B), CMC/Gel/ hAAM (C), CMC/Gel/S1P/ hAAM (D), and Histomorphometric analysis of re-epithelialization of wounds with and without scaffolds (E)

CMC: Carboxymethyl cellulose; Gel: Gelatin; EDC: 1-(3-dimethylaminopropyl)-3-ethylcarbodiimide hydrochloride; SIP: Sphingosine 1-phosphate; hAAM: Human acellular amniotic membrane

To make our *in vitro* studies even more reliable, we created a full-thickness excisional wound model in rats to see how well the CMC/Gel hydrogel with S1P heals wounds when it is covered with hAAM. Wound healing was evaluated by contraction and decrease in the size of the wound in the experimental groups: A) Negative control, B) hAAM, C) CMC/Gel/hAAM, and D) CMC/Gel/S1P/hAAM on days 4, 7, and 14 after surgery (Figure 8). Note that no sign of infection was detected in any of the treated groups. We observed 97.6% and 97.62% rates of wound closure in the CMC/Gel/hAAM and CMC/Gel/S1P/hAAM groups, respectively, which were comparable to the control group (no treatment) after 14 days. However, the rate of wound healing was higher in hAAM-treated groups (98.19%) compared to other groups.

*Histomorphometric analysis*

Figure 8E illustrates the histomorphometric assessment outcomes. The hAAM and CMC/Gel/S1P/hAAM groups were found to have the most favorable re-epithelialization and angiogenesis, with blood vessel formation being particularly heightened in the hAAM group. Our data suggests that hAAM is more efficient in promoting wound healing compared to other treatments.

*Histopathological results*

**Figure 9:**
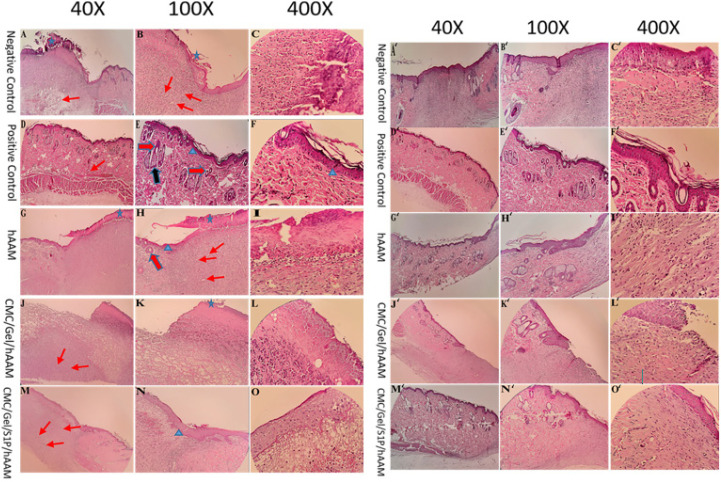
H&E staining of skin tissue at the dorsal skin of rats 7 (left, A-O) and 14 days (right, A'-O') after surgery for negative and positive control groups, and groups treated with different scaffolds

CMC: Carboxymethyl cellulose; Gel: Gelatin; hAAM: Human acellular amniotic membrane

**Figure 10:**
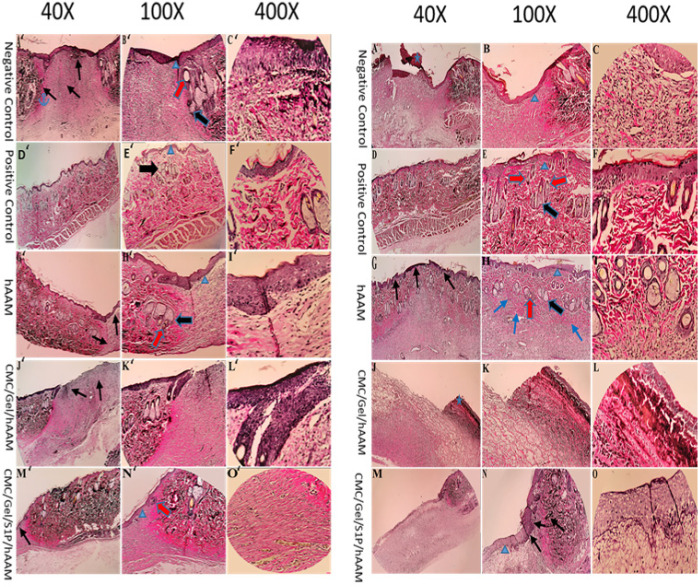
VVG staining of skin tissue at the dorsal skin of rats 7 (left, A-O) and 14 days (right, A'-O') after surgery for negative and positive control groups, and groups treated with different scaffolds

VVG: Verhoeff-Van Gieson; CMC: Carboxymethyl cellulose; Gel: Gelatin; hAAM: Human acellular amniotic membrane

Figure 9 A-O presents the histopathological findings of all groups. On day 7, in NC, hAAM, and CMC/Gel/hAAM groups, a crusty scab layer covered the wound (Figure 9 G, K, K- blue star). The hAAM and CMC/Gel/S1P/hAAM treated group presented with early epidermal proliferation and re-epithelialization (Figure 9-H, K – blue arrowhead). Blood vessel formation was significantly higher in the hAAM and CMC/Gel/S1P/hAAM treated group when compared to other groups (Figure 9-I, L-red thin arrow). Figure 10 A-O displays the VVG study findings of all groups. The formation of elastin fibers (black thin arrow) was greater in the hAAM and CMC/Gel/S1P/hAAM groups than in other groups. The mature collagen formation in the hAAM group was higher than in other treated groups (Figure 10 L-blue thin arrow).

Figure 9 A’-O’ displays the H&E study results for all groups on the 14^th^ day. The hAAM group exhibited superior wound regeneration compared to the other groups, as evidenced by complete formation and remodeling of the epidermal layer (Figure 9 L’- blue arrowhead). Additionally, blood vessel formation was more pronounced in animals treated with hAAM compared to other groups (Figure 9 I’ -red thin arrow). Animals treated with hAAM demonstrated similarities to a positive control (normal skin tissue), with rejuvenation of hair follicles, sebaceous glands, and typical epidermis (Fig 9- L’, K’ thick red and black arrow). Furthermore, collagen fiber synthesis, deposition, and maturation rates were highest in the hAAM group (Figure 9- L’, K’ thin blue arrow). On the 14^th^ day, histological analysis of VVG stained sections revealed that NC and hAAM groups had greater elastin fiber synthesis (Figure 10 – L’ thin black arrow).

***Collagen type I, IGF, TGF-beta 1, VEGF, and VCAN expression comparison***

**Figure 11:**
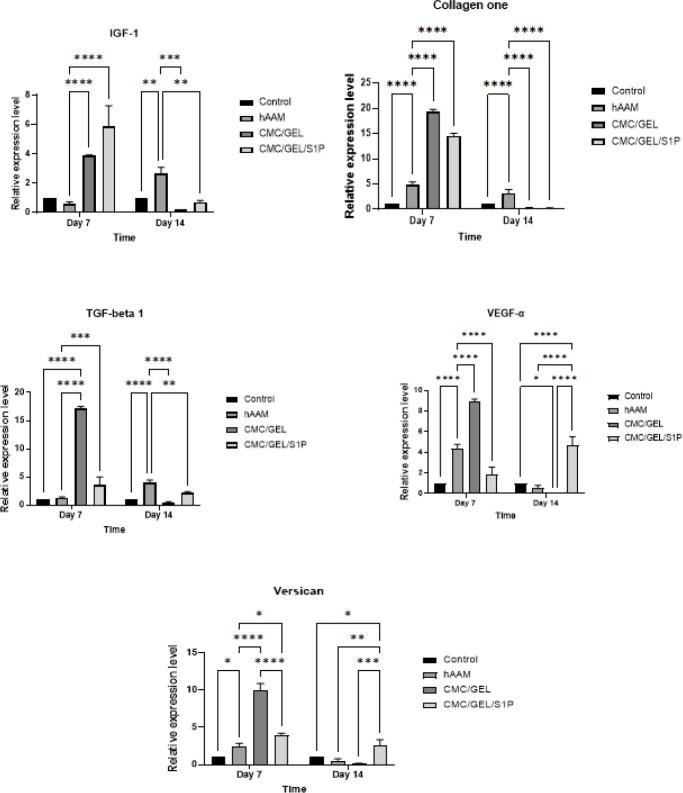
Comparing the expression of IGF I, Collagen I, TGF-beta 1, VEGF-α, and Versican genes in the control group with the treated groups; CMC/Gel/hAAM, CMC/Gel/S1P/hAAM, and hAAM, on the 7^th^ and 14^th^ day

Several growth factors such as Collagen type 1, IGF-1, TGF-, VEGF-α, and VCAN have been shown to be involved in the wound healing process. In order to explore the molecular mechanism of hydrogel CMC/Gel/S1P/hAAM in healing full-thickness skin wounds, the expression of these growth factors was measured at each stage of the wound healing process (Figure 11). qPCR analysis showed that the expression of all these growth factors was significantly up-regulated in CMC/Gel/hAAM and CMC/Gel/S1P/hAAM -treated groups compared to hAAM-treated and control groups at day 7 post-surgery. Collagen type 1 level in the hydrogel CMC/Gel/hAAM group was 19 times (the highest expression among all the groups) higher than the control group at day 7, while it was 5 and 3 times higher in the hAAM-treated group compared to the control group at days 7 and 14, respectively. The relative expression of collagen type 1 at day 14 decreased to less than 0.3 in both the CMC/Gel/hAAM and CMC/Gel/S1P groups. IGF-1 levels showed an approximately 4–6 times increase in CMC/Gel/hAAM and CMC/Gel/S1P/hAAM -treated groups at day 7 in comparison with the control group; however, there was a decreasing trend at day 14. Interestingly, the expression level of IGF-1 in the hAAM-treated group elevated almost three times from day 7 to day 14. The expression level of TGF-beta-1 was also significantly increased in the CMC/Gel/hAAM -treated group compared to all the other groups at day 7, but reduced to the least expression at day 14. At day 7, the expression level of VEGF-α was 4, 9, and 2 times higher in hAAM, CMC/Gel/hAAM, and CMC/Gel/S1P/hAAM-treated groups versus the control group, respectively. At day 14, its expression level reached about 2.6 times higher in the CMC/Gel/S1P group; however, it decreased in the CMC/Gel/hAAM and hAAM groups when compared to the control group. Finally, there was a 4-fold increase in the expression of versican in the CMC/Gel/hAAM group compared to the hAAM-treated group at day 7, followed by a remarkable reduction at day 14. In general, gene expression studies demonstrate that the use of S1P in CMC/Gel/hAAM hydrogel and the application of hAAM as a wound covering are effective in regulating gene expression of growth factors involved in wound healing. 

## Discussion

The process of wound healing involves a complex interplay of several cells and molecules, including cytokines, chemokines, growth factors, and extracellular matrix components (28). Biomaterials are utilized as scaffolds to promote cellular adhesion, proliferation, and differentiation for tissue regeneration in tissue engineering. However, various considerations must be made to ensure the optimal performance of scaffolds in tissue engineering, such as their biocompatibility, biodegradability, mechanical properties, architecture, and manufacturing technology (21, 24).

hAAM, composed of an epithelial cell layer, basement membrane, and acellular compact layer, forms the innermost layer of the placental membranes. Due to its abundance of collagen and low immunogenicity, this natural material has been used as a temporary wound dressing to reduce inflammation, promote epithelialization, and prevent scarring (1). This study aimed to assess the potential role of CMC, gelatin, S1P, and hAAM in promoting wound healing. First, human amniotic membrane cells were effectively removed from the matrix, as confirmed by the lack of residual DNA following staining with H&E and DAPI. Histological tests further validated cell removal, while the resulting matrix remained intact (30). The results of the MTT assay revealed that crosslinker EDC, at its lowest percentage, was the optimal choice for making the hydrogel. Furthermore, S1P (40 µl) was found to significantly enhance the cell viability by 116.8% and 127% after 24 and 72 hr, respectively. 

The morphological features of our CMC/Gel/S1P hydrogel scaffolds determined an optimal porosity and pore size that are suitable for cell adhesion, migration, and proliferation. The porous structure of the scaffold provides an appropriate environment for cellular attachment and proliferation by facilitating the presence of sufficient nutrients and oxygen. Additionally, SEM images and mechanical tensile tests revealed that hAAM tissue retains its structural integrity and strength even after undergoing decellularization, making it a promising candidate as a natural wound dressing. 

Our study also applied weight loss, swelling ratio, blood compatibility, blood clotting index, and FTIR tests to determine the optimal concentration of EDC crosslinker and S1P for our scaffolds. The weight loss test proved an inverse correlation between the concentration of EDC and weight loss percentages for CMC/Gel hydrogel. After 48 hr, the two least concentrated crosslinkers exhibited more than 50% weight loss, with 52.42% for EDC 0.01% and 56.92% for EDC 0.05%. Additionally, the incorporation of S1P at optimal concentrations into CMC/Gel containing 0.01% EDC enhanced scaffold degradation by 63.03% after 48 hr. This improvement enhances the feasibility and efficacy of our scaffold in daily use and replacement settings. Additionally, the swelling ratio of hydrogels initially experienced a slight increase upon the addition of crosslinker, ranging from 0.01% to 0.05%, but subsequently declined as more crosslinker was added to the hydrogel. Conversely, when incorporating S1P, the swelling ratio significantly increased. 

The properties of blood compatibility and blood clotting index revealed promising results, as CMC/Gel/EDC hydrogel with a crosslinker exceeding 0.1% showed 5% higher hemocompatibility. However, all other concentrations demonstrated hemocompatibilities lower than 5% and were in close proximity to each other. Furthermore, adding S1P to hydrogel led to a reduction in hemocompatibility from 2.40% to 2.26%. The blood clotting index (12%) of all CMC/Gel/EDC groups increased up to 19.86% after adding S1P to the hydrogel.

FTIR spectroscopy can facilitate the identification of molecular interactions between gelatin, CMC, EDC, and S1P within the scaffold. The presence of a broad peak in the 3305.58 cm^-1^ region is indicative of intermolecular interactions between the hydroxyl groups of gelatin and carboxyl groups of CMC. This phenomenon leads to a reduction in the number of available hydrogen bonds that can interact with free hydroxyl groups. Furthermore, analysis of the peak located between 1630 and 1680 cm^-1^ (represents the amide-I band) revealed that increasing the dose of CMC within hydrogel leads to conformational changes in gelatin polypeptide chains by decreasing the number of single-helices, random coils, and disordered structures within the scaffold. The addition of CMC is believed to have caused a disruption in the intermolecular hydrogen bonds between the carboxylic groups, enhancing both antisymmetric and symmetric vibrations of C=O and C-O bonds. The peak at 1413 cm^-1^ corresponds to the COO-group vibrations present in CMC. The amide-III bands, which indicate the vibrations in the plane of C-N and N-H groups of bound amides, were observed at 1323.46 cm^-1^ as anticipated. The (C-H) stretching from alkyl groups was identified by peaks at 2917.96 cm^-1^ for S1P. The broad bands between 1000-1200 cm^−1^ were due to the sugar ring absorption of the CMC molecule (31-34). 

Our research findings were reinforced by the results of H&E and VVG staining, which indicated that the viability and proliferation potential of fibroblasts displayed positive traits in rats treated with our scaffolds (hAAM, CMC/Gel/hAAM, and CMC/Gel/S1P/hAAM). It is worth noting that hAAM is a promising choice for therapeutic use, especially full-thickness skin injury, due to its accessibility, easy procurement, affordability, and low immunogenicity (24). Furthermore, hAAM offers more advantages in the maintenance of hemostasis, acceleration of wound healing, and pain relief, as well as prevention of bleeding, infection, and scar formation (27). The histological examinations demonstrate that the hAAM group exhibits greater improvement in wound healing compared to the other groups. Additionally, the positive impact of S1P in the CMC/Gel hydrogel is obvious. The analysis of gene expression further supports these findings.

Type I collagen is essential for wound healing and tissue regeneration, with an increase in synthesis during the late stages of wound healing (35). IGF-1 plays an important role in wound healing through its ability to stimulate the proliferation and migration of keratinocytes, endothelial cells, and fibroblasts (36). TGF-beta 1 stimulates cell proliferation and regulates extracellular matrix (ECM) synthesis, such as collagen (37). VEGF regulates multiple pathways during wound healing including angiogenesis, re-epithelization, and collagen synthesis (38). Versican (VCAN) is a proteoglycan composed of chondroitin sulfate and dermatan sulfate, which is found in the extracellular matrix. Its expression is significantly elevated in tissues during both developmental processes and pathological remodeling. VCAN accumulation in the wound facilitates wound repair (39).

The increases in the levels of collagen-I expression at day 7 in all treated groups in comparison to the control group are important factors for the initiation of the regeneration phase and exhibit a great impact on the healing process at this stage. At day 14, the regeneration process is still ongoing, and the higher level of collagen type I compared to the control group exhibits a promising effect for wound healing at a late stage (35). This collagen-I up-regulation at day 14 in the hAAM group is in agreement with our histopathological findings. Generally, the increase in IGF-I level at each stage of wound healing can be a plus point (40). On day 7, the IGF-I level increased significantly in CMC/Gel/hAAM and CMC/Gel/S1P/hAAM groups rather than the control group, and at day 14, there was an up-regulation of IGF-I in the hAAM group, which again was in agreement with our histopathological studies. The higher expression of IGF-I in hydrogel CMC/Gel/hAAM and CMC/Gel/S1P/hAAM groups at earlier phases of wound healing recruited more fibroblasts and strongly contributed to fibroblast proliferation during wound healing, resulting in better wound closure for the hydrogel group. The increase in TGF-beta 1 levels at different treatments advances the healing process by stimulating wound contractions (41). At day 7, there was up-regulation in TGF-beta 1 levels in all treated groups rather than the control group, with the highest amount in the CMC/Gel/hAAM group. At day 14, there were also increases in TGF-beta 1 expression in hAAM and CMC/Gel/S1P/hAAM groups compared to the control group, which regulates the function of keratinocytes, fibroblasts, endothelial cells, monocytes, and other cell types (41). The up-regulation of VEGF-α plays an important role in the healing process by stimulating angiogenesis, which causes proliferation and re-epithelialization (42). At day 7, all of the treatment groups increased significantly more than the control group, and this improvement continued in the CMC/Gel/S1P/hAAM group at day 14 which is due to the angiogenic effect of S1P. Reducing VEGF-α at day 14 in hAAM and CMC/Gel/hAAM groups could be beneficial for scar tissue formation restrictions (42). Additionally, the descending trend of VEGF-α expression in hAAM and hydrogel CMC/Gel/hAAM groups during wound healing synchronized with the physiological wound healing process, and its relatively higher level accelerated the process (43). At day 7, there was a general increase in VCAN expression in all treated groups rather than the control group, which was in agreement with the expression of TGF-beta 1. These findings show that up-regulation of VCAN facilitates the healing process (39). At day 14, the hAAM and CMC/Gel/hAAM groups show potential in prevention of scarring by decreasing the expression of VCAN.

The expression levels of all genes (COLLAGEN, IGF, VEGF, VERSICAN, and TGF-beta 1) in the CMC/Gel/hAAM hydrogel group increased on day 7 and then decreased on day 14. However, when S1P was added to the CMC/Gel/hAAM hydrogel, there was an improvement in wound healing and gene expression, which was consistent with our histopathological studies. Taken together, these results revealed that the hAAM and hydrogel CMC/Gel/S1P/hAAM groups promoted the wound healing process by up-regulating growth factors levels, like Collagen type 1, IGF-I, TGF-beta 1, VEGF-α, and VCAN, and then recruiting fibroblasts and promoting ECM synthesis, collagen deposition, and granulation tissue thickness. 

The results presented in our study are consistent with past research and confirm those findings, as in a study, the effect of injectable S1P on the healing of skin wounds in diabetic rats has been investigated (16). Just as the formation of new blood vessels was observed in the *in vivo* results of our study, the results of this research also proved that S1P significantly accelerates the healing of skin wounds with the process of new blood vessel formation and affects all the main cellular steps responsible for wound healing. This study also emphasizes the unique potential of this molecule in the treatment of diabetic wounds, especially as an angiogenic agent in the treatment of wounds (16). Other research results also confirm these findings and prove a five-fold increase in wound contraction caused by granulation and acceleration of diabetic wound closure using S1P (44). The role of S1P as a new therapeutic molecule with anti-scar effect in surgery and chronic wound management in another investigation confirmed the results obtained in our study (18). In another study, a decellularized human amniotic membrane (hAAM) was used to create a skin replacement model to create a scaffold for keratinocyte cell culture. Evaluating the effectiveness of this skin substitute by histological, immunohistochemical, and immunofluorescent methods shows results similar to our studies. In this way, hAAM can be a suitable matrix to support the adhesion of cells, and it provides the possibility of obtaining a complete and integrated scaffold for the creation of a skin substitute, and it can also be suggested as a suitable skin dressing in clinical use (45). Also, in a similar study, hAAM was used to make a tissue-engineering skin substitute for the implantation of human embryonic fibroblasts, this proposed skin substitute proved to accelerate wound healing (46).

In a study, hydrogel films composed of low methoxyl pectin (LMP), gelatin, and carboxymethyl cellulose (CMC) were fabricated. After evaluating this hydrogel film, the researchers suggested it as an effective dressing with high liquid absorption capacity, and the ability to retain liquids and transfer water vapor at a suitable speed. These results are aligned with our findings about synthesized gelatin/CMC hydrogel **(**47**)**. In a study using CMC sheets along with silver nanoparticles, antibacterial and wound-healing properties were investigated. The results, in accordance with our findings, have introduced carboxymethyl cellulose (CMC) as an effective substance for healing wet wounds. These results prove the importance of CMC and its therapeutic potential as a wound dressing (48). These findings open up the possibilities for future investigations in this area.

**Table 1:** Sequence of primers used in Real-Time PCR in tissue examination

Gene	Forward primer	Reverse primer	Product Length	Annealing Temperature
GAPDH	TCTCTGCTCCTCCCTGTTCTA	ATGAAGGGGTCGTTGATGGC	177	61 °C
VCAN	CCCCTGCAACTACCACCTCACC	TCTTTCCAAAGGTCTTGGCATTTTCT	95	62 °C
IGF-1	GCTTTTACTTCAACAAGCCCACA	TCAGCGGAGCACAGTACATC	129	61 °C
VEGF-α	CCAGGCTGCACCCACGACAG	CGCACACCGCCATTAGGGGCA	179	62 °C
TGF-β	AAGAAGTCACCCGCGTGCTA	TGTGTGATGTCTTTGGTTTTGTCA	70	61 °C
COL1A1	CATGTTCAGCTTTGTGGACCT	GCAGCTGACTTCAGGGATGT	94	61 °C

**Table 2:** Mechanical properties of human amniotic membrane and decellularized human amniotic membrane

Mechanical properties	Amniotic membrane (control)	Acellular amniotic membrane
Thickness	0.01 mm	0.01 mm
Elongation	Peak:6.58% Break:6.60%	Peak:22.82% Break:22.83%
Young's modulus	228.73 Mpa	132.23 MPa

CMC: Carboxymethyl cellulose; Gel: Gelatin; EDC: 1-(3-dimethylaminopropyl)-3-ethylcarbodiimide hydrochloride

EDC: 1-(3-dimethylaminopropyl)-3-ethylcarbodiimide hydrochloride

*: *P*-value<0.05, **: *P*-value<0.01, ***: *P*-value<0.001, and ****: *P*-value<0.0001

MTT: 3-(4, 5- dimethylthiazol-2-yl)-2,5 diphenyl tetrazolium bromide; CMC: Carboxymethyl cellulose; Gel: Gelatin; EDC: 1-(3-dimethylaminopropyl)-3-ethylcarbodiimide hydrochloride

Most of the treated groups showed an increase in gene expression on the 7th day compared to the control group, but the gene expression on the 14th day in the treated groups showed different results. (*: *P*<0.05, **: *P*<0.01, ***: *P*<0.001, and ****: *P*<0.0001).

## Conclusion

Our study revealed that a hydrogel made from CMC/Gel/EDC and containing S1P, with a hAAM as a covering, had a favorable impact on wound healing. The histoarchitecture of the hAAM matrix remained undisturbed, and our findings indicated that the decellularization process did not negatively affect the key components of the tissue. While not central to our study, we aimed to construct a hydrogel scaffold with essential characteristics for topical use and tissue-engineered human amniotic membrane. The CMC/Gel/EDC/S1P hydrogel with hAAM covering facilitated dermal cell proliferation and migration and promoted blood vessel formation in histopathological tests. Furthermore, its physical properties demonstrated promising qualities as a biocompatible and biodegradable wound dressing. Thus, this hydrogel demonstrated significant potential for enhancing wound healing.

## Data Availability

The datasets generated and analyzed for this study are available from the corresponding author upon reasonable request.
